# CAFC-Net: A Critical and Align Feature Constructing Network for Oriented Ship Detection in Aerial Images

**DOI:** 10.1155/2022/3391391

**Published:** 2022-02-24

**Authors:** Dongdong Zhang, Chunping Wang, Qiang Fu

**Affiliations:** Department of Electronic and Optical Engineering, People Liberation Army Engineering University-Shijiazhuang, Shijiazhuang, Hebei 050003, China

## Abstract

Ship detection is one of the fundamental tasks in computer vision. In recent years, the methods based on convolutional neural networks have made great progress. However, improvement of ship detection in aerial images is limited by large-scale variation, aspect ratio, and dense distribution. In this paper, a Critical and Align Feature Constructing Network (CAFC-Net) which is an end-to-end single-stage rotation detector is proposed to improve ship detection accuracy. The framework is formed by three modules: a Biased Attention Module (BAM), a Feature Alignment Module (FAM), and a Distinctive Detection Module (DDM). Specifically, the BAM extracts biased critical features for classification and regression. With the extracted biased regression features, the FAM generates high-quality anchor boxes. Through a novel Alignment Convolution, convolutional features can be aligned according to anchor boxes. The DDM produces orientation-sensitive feature and reconstructs orientation-invariant features to alleviate inconsistency between classification and localization accuracy. Extensive experiments on two remote sensing datasets HRS2016 and self-built ship datasets show the state-of-the-art performance of our detector.

## 1. Introduction

Ship detection is a technology aiming at distinguishing and locating ships of interest. It is widely used in port management, cargo transportation, maritime rescue, and defense buildup [1]. Ships are characterized by their large-scale variation, large aspect ratio, and dense arrangement, making them more difficult to detect than other objects [2, 3], such as aircrafts [4, 5], vehicles [6, 7], and buildings [8]. Although a number of traditional methods [9–12] have been developed to identify ships and much progress has been made, they still suffer from limitations in efficiency, robustness, performance, and more.

In recent years, CNN has emerged as a mainstream approach to support target detection. With the powerful feature extraction capability of CNN, CNN-based methods can deliver much improved target detection performance. The region-based convolutional neural network (RCNN) [13] generates region proposals by selective search, extracts features with CNN, and classifies targets by the SVM linear classifier. Despite its obvious limitations in speed and storage space, RCNN offers better detection performance than traditional detection methods. Fast-RCNN [14] is an enhanced version of RCNN. It leverages shared computation to help improve the detection efficiency and reduce the space required for storage. Faster-RCNN [15] is a further improvement based on Fast-RCNN. It uses CNN to generate region proposals and can yield much higher detection speeds by running the whole detection process on the GPU. Although these methods have achieved great success, their performance will decline sharply when they are used for ship detection in aerial images. This is because they have to deal with the following three challenges. (1) Large-scale variation: the ships in aerial images often vary greatly in their scale; (2) large aspect ratio: ships are usually long strips with a large aspect ratio; and (3) dense arrangement: ships for detection are often densely arranged.

To resolve these challenges, a common practice is to prepare a large number of anchor boxes with different angles, scales, and aspect ratios, from which a sufficient number of high-quality anchor boxes can be obtained to help improve the detection performance. However, excessive prior anchor boxes can lead to two problems as follows.

**Figure 1:**
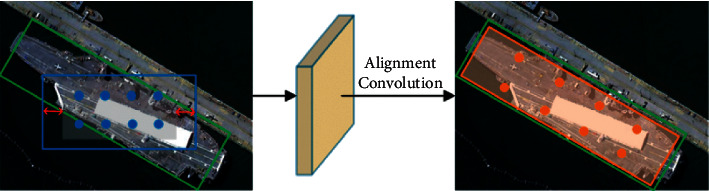
Misalignment problems in single-stage detectors.

*Slowed Detection Speed*. Many useless anchor boxes lead to severely redundant calculation, which will inevitably slow down the detection speed. In contrast with RCNN-based detectors, single-stage detectors directly regress bounding boxes on feature maps without going through the proposal region generation stage. The architecture offers high computational efficiency and can therefore solve the speed problem. However, single-stage detectors are plagued by misalignment problems [16] (Figure 1), making them perform poorly in accuracy.

**Figure 2:**
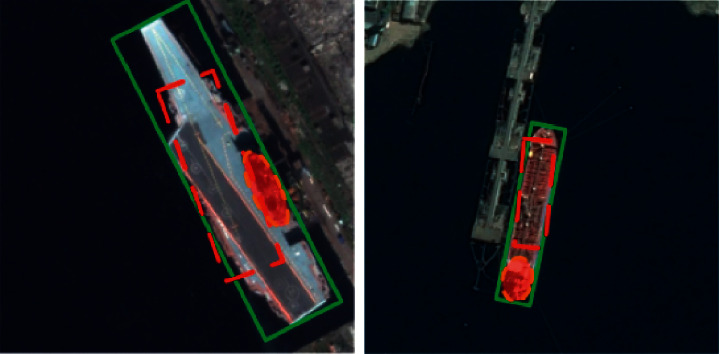
Variations between different boxes.

*Impaired Accuracy*. As shown in Figure 2, there is an intersection over union (IoU) larger than 0.5 between the anchor boxes, whether they are in the color of green or red, and the GT boxes, so these anchor boxes are considered as positive samples. However, the red anchor boxes do not capture any critical features necessary for ship detection. Despite their high localization accuracy, these anchor boxes reduce the detection performance and should preferably be discarded. The inconsistency between the training sample division and the regression results will further lead to a gap between the classification scores and localization accuracy of the detection. Furthermore, the critical features for regression and classification need to be extracted separately. These findings agree with those of some previous studies [17, 18]. The anchors having the ability to capture the critical features of the objects are key to detecting objects in remote sensing imagery.

To address the misalignment problem in single-stage detectors and extract critical features for detection, we propose a Critical and Align Feature Constructing Network (CAFC-Net) consisting of three modules: a Bias Attention Module (BAM), a Feature Alignment Module (FAM), and a Distinctive Detection Module (DDM). The BAM can generate different critical features for classification and regression tasks. The FAM employs an Anchor Optimization Network (AON) and an Alignment Convolution (ACL). Based on the critical features for regression tasks, the AON allows high-quality anchor boxes to be generated. Unless other methods with dense prior anchor boxes, our method employs only one squared anchor box for each location in the feature map and, with the help of the AON, refines them into high-quality rotated anchor boxes. Then, the ACL, a variant of deformable convolution, automatically aligns features according to the shapes, sizes, and orientations of the corresponding anchor boxes. In the DDM, orientation-invariant features can be generated by pooling orientation-sensitive features, and then orientation-invariant features can be reconstructed with biased critical features for classification. Finally, we feed the orientation-sensitive features and the reconstructed orientation-invariant features into a regression subnetwork and a classification subnetwork to produce final predictions. Extensive experiments on the HRSC2016 dataset and a self-built ship dataset demonstrate that our proposed method can deliver strong ship detection performance.

The main contributions of this paper are summarized as follows:

1. Biased attention is proposed to construct different critical features for various tasks. Biased critical features provide more useful semantic information for individual tasks, facilitating accurate classification and location.
2. For the misalignment between axis-aligned convolution features and arbitrarily oriented objects, we propose a new Alignment Convolution. The AlignConv costs almost same amount of time compared with standard convolution and can be easily embedded in many detectors.
3. Through constructing DDM, inconsistency between classification and regression can be bridged.

With the BAM and the FAM embedded, we design a light single-stage detector which can generate biased critical features for classification and regression and align features for accurate ship detection in aerial images.

The rest of this paper is organized as follows. In Section 2, we briefly review previously published studies on ship detection. Section 3 describes in detail the proposed method. Section 4 presents the experiments on two aerial image datasets and the analysis process. Finally, Section 5 concludes the paper with a discussion on the results.

## 2. Summary Review of Previous Studies

**Figure 3:**
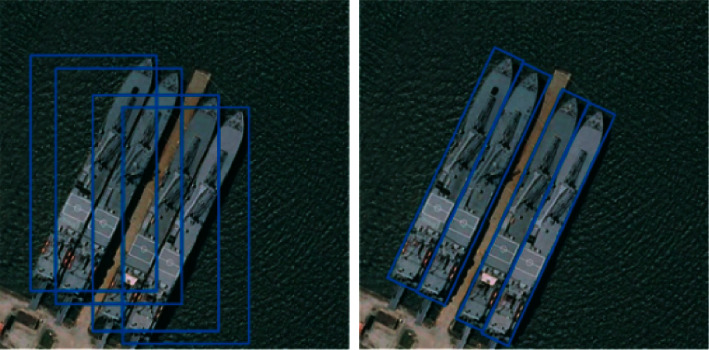
Schematic illustration of HBB and RBB.

Traditional methods for ship detection in SAR images [19–21] perform sea-land segmentation and then object detection and recognition based on manually produced features. However, such methods have high false alarm probability, consume a large amount of time, and perform poorly for detection in nearshore areas. With the advancement of CNN, a series of CNN-based ship detection frameworks have been reported [22–25]. Inspired by the idea of deep networks, Zhang et al. [22] presented a faster region-based CNN method to detect ships from high-resolution remote sensing imagery. Huang et al. [23] proposed a novel network architecture named Squeeze Excitation Skip-connection Path Networks (SESPNets) to improve feature extraction capability and used soft nonmaximum suppression to raise detection accuracy. However, these methods are based on the Horizontal Bounding Box (HBB). Due to the arbitrary orientation and large aspect ratio of ships, HBB-based detectors often produce a lot of redundant information, which may mislead the detection, as shown in Figure 3(a). To alleviate this problem, some studies have tried to use the Rotated Bounding Box (RBB) (Figure 3(b)) to locate ships [26–28]. For instance, Liu et al. [26] proposed a Rotated Region-based CNN (RRCNN) to predict the arbitrary direction of ships. Zhang et al. [27] utilized a Rotation Region Proposal Network (RRPN) and a Rotation Region of Interest (RRoI) pooling layer to detect arbitrarily oriented ships. However, to ensure high overlaps with the rotated objects, these methods adopted densely arranged anchor boxes with different angles, scales, and aspect ratios. Most of the anchor boxes have no intersection with the targets, which brings heavy computation and the severe imbalance problem. Some studies addressed these challenges by setting fewer anchor boxes without compromising the detection performance [29, 30]. Ding et al. [29] proposed a RoI Transformer to transform a horizontal RoI into a rotational RoI by spatial transformation, which eliminated the need for a large number of prior anchor boxes and alleviated the misalignment problem. R^3^Det [30] achieved state-of-the-art performance by re-encoding the position information to align the features of horizontal anchor boxes. In contrast with the above methods, the proposed CAFC-Net can generate high-quality anchor boxes by refining horizontal anchor boxes into rotated anchor boxes, improving the detection speed with a reduced number of anchor boxes.

The detection by an object detector usually involves two parallel tasks: object classification and bounding-box regression, which share the same features from the backbone network. However, classification and regression based on shared features do not deliver satisfactory performance. Liao et al. [31] observed that sharing features would degrade the performance due to the inconsistency between classification and regression tasks, so they constructed rotation-invariant and rotation-sensitive features for each. Wu et al. [32] proposed to construct different head architectures for different tasks, i.e., fully connected head for classification and convolution head for regression. Inspired by [18, 33], we build the BAM to extract biased critical features and use them for classification and regression tasks, respectively.

## 3. Proposed Method

**Figure 4:**
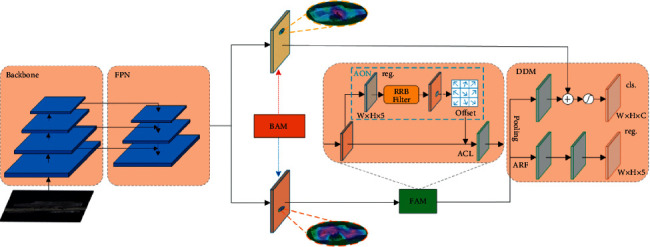
Architecture of the proposed CAFC-Net.

The overall architecture of the proposed CAFC-Net is shown in Figure 4. First, we use RetinaNet [34] as the baseline of our network. Different critical features are constructed for classification and regression through the BAM. Then, the AON can generate high-quality anchor boxes based on the critical regression features. Next, the ACL can align features according to the corresponding anchor boxes. Finally, through the DDM, the features used for classification are reconstructed, which are different from those used for regression. In this way, the inconsistency between classification and regression can be alleviated and the detection performance can be effectively improved. The architecture of the proposed CAFC-Net is described in detail as follows.

### 3.1. RetinaNet

RetinaNet consists of a backbone network and task-specific subnetworks. A feature pyramid network (FPN) [35] is adopted as the backbone network to extract multiscale features. Both the classification and regression subnetworks are convolutional networks. Moreover, focal loss is used to address category imbalance caused by excessive background during training.

RetinaNet is suitable for multiclass object detection, for which four parameters (*x*, *y*, *w*, *h*) are used to locate an HBB. To facilitate the detection of arbitrarily oriented ships, five parameters (*x*, *y*, *w*, *h*, *θ*) are used to represent rotation anchor boxes. Lying in the range of [−*π*/2,0), *θ* denotes the acute angle between the box and the *x*-axis, and for the other side, we denote it as *h*. Therefore, we add an angular offset to the predictive regression subnet, which is represented as follows:

(1) $$\begin{array}{c} d_{x}=\frac{\left(x-x_{a}\right)}{w_{a}},\\ d_{y}=\frac{\left(y-y_{a}\right)}{h_{a}},\\ d_{w}=\log\left(\frac{w}{w_{a}}\right),\\ d_{h}=\log\left(\frac{h}{h_{a}}\right),\\ d_{\theta }=\theta -\theta_{a},\\ d_{x}^{*}=\frac{\left(x^{*}-x_{a}\right)}{w_{a}},\\ d_{y}^{*}=\frac{\left(y^{*}-y_{a}\right)}{h_{a}},\\ d_{w}^{*}=\log\left(\frac{w^{*}}{w_{a}}\right),\\ d_{h}^{*}=\log\left(\frac{h^{*}}{h_{a}}\right),\\ d_{\theta }^{*}=\theta^{*}-\theta_{a}, \end{array}$$

where *y*, *w*, *h*,  and *θ* indicate the center coordinates, width, height, and angle of the box, respectively. Variables *x*, *x*_*a*_,  and *x*^*∗*^ represent the predicted box, anchor box, and ground-truth box, respectively.

### 3.2. Biased Attention Module (BAM)

**Figure 5:**
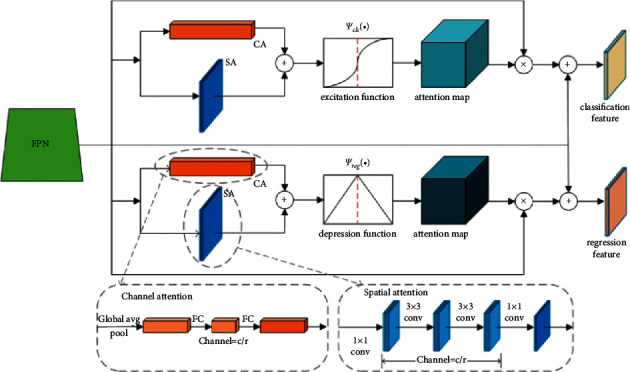
Schematic illustration of the BAM.

The overall structure of the BAM is shown in Figure 5. For classification, we tend to select significant global features to reduce noise interference. For regression, we pay more attention to the features of object boundaries and less to those of irrelevant regions.

Given input feature *F* ∈ *R*^*C*×*H*×*W*^, the BAM constructs biased features as follows:

(2) $$\begin{array}{c} M\left(F\right)=\psi \left(M_{C}\left(F\right)+M_{S}\left(F\right)\right),\\ F^{\prime }=F+F⊗M\left(F\right), \end{array}$$

where ⊗ denotes element-wise multiplication.

First, we extract channel attention map *M*_*C*_ and spatial attention map *M*_*S*_ through two separate branches. The channel attention network can assign different weights to each channel according to its contribution to the detection task. The weight of each channel is extracted by global average pooling and fully connected layers. The channel attention is computed as follows:

(3) $$\begin{array}{c} M_{C}\left(F\right)=\sigma \left(W_{1}\left(W_{0}\text{AvgPool}\left(F\right)\right)\right), \end{array}$$

where *W*_0_ ∈ *R*^*C*/*r*×*C*^ and *W*_1_ ∈ *R*^*C* × *C*/*r*^ are the weights and *σ* represents the sigmoid function.

Correspondingly, spatial attention is applied to establish the dependencies between pixels of the input image. It can be computed as follows:

(4) $$\begin{array}{c} M_{S}\left(F\right)=\sigma \left(f_{3}^{1\times 1}\left(f_{2}^{3\times 3}\left(f_{1}^{3\times 3}\left(f_{0}^{1\times 1}\left(F\right)\right)\right)\right)\right), \end{array}$$

where *f* denotes a convolution operation and the superscripts denote the convolution filter size. There are two 1×1 convolutions for channel reduction. The intermediate 3×3 dilated convolutions are applied to expand the receptive field.

Next, by combining the channel attention *M*_*C*_(*F*) and the spatial attention *M*_*S*_(*F*), we get attention response map *M*(*F*). Due to their different shapes, the two attention maps are expanded to *R*^*C*×*H*×*W*^ before being combined. Furthermore, we build powerful biased critical features through different functions *ψ*(•). For classification, we exploit an excitation function to focus the attention of the detector on significant parts of the objects. The excitation function is defined as follows:

(5) $$\begin{array}{c} \psi_{cls}\left(x\right)=\frac{1}{1+e^{-\left(x-0.5\right)}}. \end{array}$$

Since the significant areas of critical classification features are sufficient for accurate classification, there is no need to attend to additional information. Therefore, the effects of the significant areas are excited, while the irrelevant features with an attention weight less than 0.5 are suppressed. In this way, the classifier can pay less attention to the irrelevant regions and reduce the risk of overfitting and missclassification.

Meanwhile, for the regression branch, the detector needs to pay more attention to the contour and context information of the objects. Thus, the following depression function is implemented:

(6) $$\begin{array}{c} \psi_{\text{reg}}\left(x\right)=\left\{\begin{array}{cc} x, & x<0.5,\\ 1-x, & \text{otherwise.} \end{array}\right. \end{array}$$

In (6), the regions with high response are suppressed, which forces the model to look for potential features for accurate localization. The curve of the function *ψ*(•) is shown in Figure 5.

**Figure 6:**
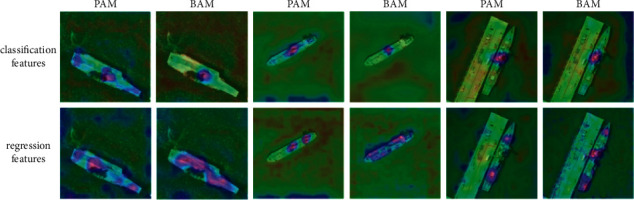
The visualization results of FAM and BAM.

Finally, biased attention maps are element-wisely multiplied with the original feature map *F* to extract critical features. As shown in (2), the original feature map *F* and the critical feature map are merged by element-wise summation to obtain powerful feature representations. The visualization results of biased critical features are shown in Figure 6. It can be seen that the BAM is capable of efficiently extracting the critical features required by different tasks. Specifically, the features used for classification are more concentrated in areas that are easy to identify, avoiding interference from other parts of the ship. Features used for regression are widely distributed across the ship, which helps to identify the boundary and thus enables more accurate localization.

### 3.3. Feature Alignment Module (FAM)

The FAM consists of an Anchor Optimization Network (AON) and an Alignment Convolution (ACL), as shown in Figure 4. In this section, we will introduce them in detail.

#### 3.3.1. Anchor Optimization Network

We take each pixel of the feature map as an anchor to generate a horizontal anchor box and adjust it through preliminary regression. Based on the IoU between the anchor box and the ground truth, an RBB filter is used to remove anchor boxes with an IoU less than 0.3. Then, the AOM is used to reduce the number of anchor boxes and generate high-quality proposals.

#### 3.3.2. Alignment Convolution Layer (ACL)

In a standard 2D convolution, we first sample over the input feature map *X* by a regular grid *R*={(*r*_*x*_, *r*_*y*_)} and then sum up the sampled values weighted by *W*. The grid *R* represents the receptive field size and dilation. For example,

(7) $$\begin{array}{c} R=\left\{\left(-1,-1\right),\left(-1,0\right),\ldots ,\left(0,1\right),\left(1,1\right)\right\}, \end{array}$$

defines a 3×3 kernel and dilation 1.

For each location *p* on the output feature map *Y*, we have

(8) $$\begin{array}{c} Y\left(p\right)=\sum_{r\in R}W\left(r\right)\cdot X\left(p+r\right), \end{array}$$

where *r* enumerates the locations in *R*.

Unlike a standard convolution, the Alignment Convolution (AlignConv) can be expressed as follows:

(9) $$\begin{array}{c} Y\left(p\right)=\sum_{r\in R;o\in \vartheta }W\left(r\right)\cdot X\left(p+r+o\right)\cdot \Delta m, \end{array}$$

where Δ*m* is a learnable modulation scalar for each location and lies in the range [0, 1]. Each modulation scalar can be viewed as a weight, and more accurate feature extraction can be achieved by assigning different weights to the offset-corrected regions. For each location, the offset field *ϑ* is computed as the difference between the anchor-based sampling position and the regular sampling position (i.e., *p*+*r*) and can be computed as follows:

(10) $$\begin{array}{c} \vartheta =\underset{r\in R}{\sum }\left(L_{p}^{r}-p-r\right). \end{array}$$

The corresponding anchor box at location *p* can be represented by five parameters (*x*, *y*, *w*, *h*, *θ*). For each *r* ∈ *R*, the anchor-based sampling location *L*_*p*_^*r*^ can be defined as follows:

(11) $$\begin{array}{c} L_{p}^{r}=\frac{1}{S}\left(\begin{array}{cc} \cos\;\theta & -\sin\;\theta \\ \sin\;\theta & \cos\;\theta \end{array}\right)\left(\left(\begin{array}{c} x\\ y \end{array}\right)+\frac{1}{k}\left(\begin{array}{c} w\\ h \end{array}\right)\cdot r\right), \end{array}$$

where *k* indicates the kernel size and *S* denotes the stride of the feature map.

In this way, the axis-aligned convolutional features *X*(*p*) of a given location *p* can be transformed into arbitrarily oriented ones based on the corresponding anchor box.

As shown in Figure 4, for an *H* × *W* × 5 anchor prediction map, we first convert the relative offset (Δ*x*, Δ*y*, Δ*w*, Δ*h*, Δ*θ*) into absolute anchor boxes (*x*, *y*, *w*, *h*, *θ*). Then, the offset field calculated by (10) along with input features is fed into the AlignConv to extract aligned features. The ACL has notable advantages in which it is a light convolutional layer and that the time it takes for offset computation is negligible.

### 3.4. Distinctive Detection Module

As shown in Figure 4, the DDM can alleviate the inconsistency between classification scores and localization accuracy. An active rotating filter (ARF) [36] is adopted to obtain orientation-sensitive features. An ARF is a *k* × *k* × *N* filter that actively rotates *N* − 1 times during convolution to produce a feature map of *N* orientation channels (*N* is 8 by default), which explicitly encodes the orientation information. Since the ARF convolves a feature map *X*, the *k* − *th* orientation output of *Y* can be computed as follows:

(12) $$\begin{array}{c} Y^{\left(k\right)}=\overset{N-1}{\underset{n=0}{\sum }}F_{\theta_{k}}^{\left(n\right)}\cdot X^{\left(n\right)},\\ \theta_{k}=k\frac{2\pi }{N},\;k=0,\ldots ,N-1, \end{array}$$

where *F*_*θ*_*k*__ is the clockwise *θ*_*k*_ − rotated version of *F* and *F*_*θ*_*k*__^(*n*)^ and *X*^(*n*)^ are the *n* − *th* orientation channel of *F*_*θ*_*k*__ and *X*, respectively. Applying the ARF to the convolution layer, we can capture rotation-sensitive feature maps and improve the generalizability of the rotating samples. Orientation-sensitive features are preferred for bounding-box regression tasks, while rotation-invariant features are required for object classification tasks. Following [36], the DDM obtains rotation-invariant features by pooling the orientation-sensitive features. The rotation-invariant features can be easily extracted by selecting the orientation channel with the strongest response as output feature *X*_pooling_.

(13) $$\begin{array}{c} X_{\text{pooling}}=\begin{array}{cc} \overset{N-1}{\underset{n=0}{\max}} & X^{\left(n\right)} \end{array}. \end{array}$$

Since the pooling operation is unordered and applied to all the *N* response maps, the resulting feature maps are locally invariant to object rotation. Then, we fuse the orientation-invariant features with biased critical features and enhance significant features by using a Sigmoid function. Through the reconstruction, the features for classification are made compatible with orientation-invariant features and biased attention. Finally, we feed the reconstructed orientation-invariant features and orientation-sensitive features into two subnetworks for classification and regression, respectively.

## 4. Experiments and Analysis

### 4.1. Datasets

In the experiments, the HRS2016 [37] dataset and a self-built dataset, called Ship, are used to verify the effectiveness of the proposed method.

*HRSC2016.* This is a high-resolution ship recognition dataset which contains 1061 images and more than 20 categories of ships in various appearances, scales, and orientations. The image size ranges from 300 × 300 to 1500 × 900. The entire dataset is divided into a training set (849 images) and a test set (212 images). Horizontal flip is adopted to enhance the training set, and all samples are resized to 800 × 800 before training and testing.

*Ship*. We collect 1002 aerial images from Google Maps to construct the Ship dataset. All the images are in the size of 600 × 1000. See Table 1 for details of the dataset. Although the Ship dataset has fewer images than HRSC2016, it contains more objects than the latter. Since the images in the Ship dataset are mostly in the background of commercial ports, commercial ships make up the majority of the images. This means that the richness of ships in the Ship dataset is not as good as that in HRSC2016.

### 4.2. Experiment Implementation

The backbone of the CAFC-Net is ResNet-50 [38], unless otherwise specified. We use a feature pyramid of *P*_2_, *P*_3_, *P*_4_, *P*_5_,  and *P*_6_ (the strides are 4, 8, 16, 32, and 64) to detect multiscale objects. For each position of feature maps, only one anchor is set to regress nearby objects.

All experiments are implemented on the TensorFlow deep learning framework and executed on a graphic workstation with dual Intel Xeon-E5 CPUs, a single NAVIDIA TITAN RTX GPU (24 GB of video memory), and 64 GB of RAM. The network is trained with an SGD optimizer. The learning rate is 0.001 and is divided by 10 at each decay step. The weight decay and momentum are 0.0001 and 0.9, respectively.

### 4.3. Ablation Studies

#### 4.3.1. Evaluation of Different Components

We conduct component experiments to validate the contributions of the proposed components.  Table 2 shows the specific settings and test results of each group of experiments. Since only one anchor box is set on each feature map, it is difficult for the baseline model to obtain enough critical features, and the mAP value is only 75.62%. With the BAM used alone, the detection performance is improved by 2.79%, indicating that the critical features constructed by the BAM can facilitate the matching of anchor boxes. With the FAM used alone, the detection performance is improved by 4.24%, indicating that the FAM can generate high-quality anchors with feature alignment. With the BAM and DDM used together, the mAP value is improved by 4.97% compared with when the BAM used alone, indicating that the DDM's ability to construct different features for two subnetworks helps to improve detection accuracy. With the BAM and FAM used together, the mAP value reaches 85.65%, which represents significantly improved detection performance. Finally, the mAP of the CAFC-Net reaches 91.18%, which is 15.56% higher than that of the baseline model, proving the effectiveness of our network.

#### 4.3.2. Evaluation of BAM

Comparative experiments are conducted to verify the effectiveness of the BAM. The results are shown in Table 3. By adding an attention mechanism, the detection performance is improved by 2.79% compared with the baseline model. The results show that the attention mechanism can make the detector focus on the target area. The detection accuracy is improved by embedding an excitation function and a depression function in the network.

To verify the effectiveness of the BAM in a more straightforward way, the results of some features of the BAM and PAM [18] are visualized in Figure 6. The heat map induced by the BAM responds to the area of task-sensitive critical features more accurately. The features required for classification should be concentrated in the target area. The BAM outperforms the FAM in eliminating the influence of irrelevant features. At the same time, the clues required for regression are more likely to be distributed on the edge of targets. The feature distribution of the BAM is more uniform than that of the FAM, which is more conducive to higher levels of localization accuracy.

#### 4.3.3. Evaluation of AlignConv

We verify the effectiveness of AlignConv by comparing it with Conv [39–41], DeformConv [42], and DeformConv v2 [43]. The experimental results are shown in Table 4. Compared with standard convolution, AlignConv improves the mAP by 3.70% while introducing a mere of 4 ms latency into the detection process. Although DeformConv and DeformConv v2 have almost no latency compared with standard convolution, they only deliver mAP values of 88.68% and 89.88%, respectively. The results show that AlignConv can effectively improve the detection performance with negligible latency.

### 4.4. Comparison with Other State-of-the-Art Methods

**Figure 7:**
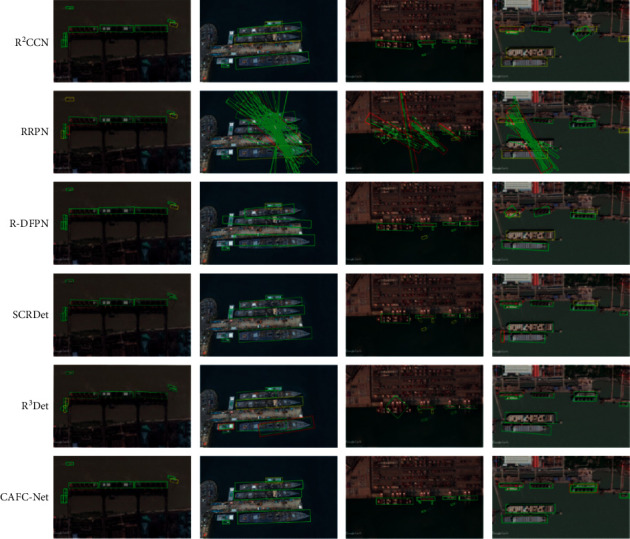
Results of detection of images in the Ship dataset.

**Figure 8:**
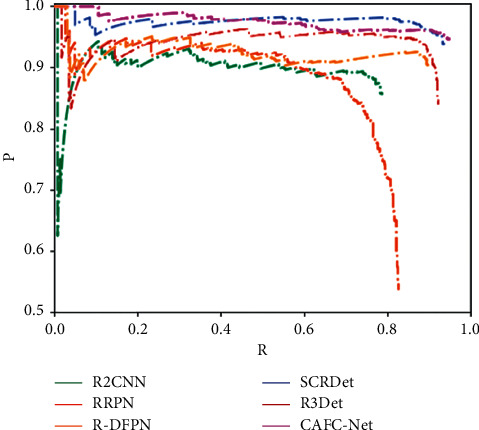
The P-R curves of different methods.

We evaluate the overall performance of the proposed network by comparing it with other state-of-the-art methods on the Ship dataset. As shown in Table 5, our proposed framework delivers an mAP value of 91.18%, which represents higher detection performance than R^2^CNN [44], RRPN [45], R-DFPN [1], SCRDet [46], and R^3^Det [30]. The qualitative detection results are visualized in Figure 7. Specifically, the green boxes are correctly detected ship targets, yellow boxes are missed targets, and red boxes are false detection targets. Compared with the above five algorithms, the CAFC-Net produces fewer false predictions and more accurate localization when detecting densely arranged ships. Besides, from the visualized mAP and performance results, we can easily see that our method is particularly effective in detecting ships with a large aspect ratio and a large-scale variation. The F-values, as shown in Table 5, and P-R curves, as shown in Figure 8, illustrate that the overall performance of the CAFC-Net is even better.

## 5. Conclusion

In this paper, we build an end-to-end single-stage rotation detector, called the CAFC-Net, to improve the performance of ship detection in aerial images. Several novel modules are designed for the model. First, we design a biased attention module, which can extract biased critical features for classification and regression. Then, high-quality anchor boxes can be generated. The features can be aligned according to the corresponding high-quality anchor boxes. Finally, we adopt a distinctive detection module which can construct different features for classification and regression to alleviate the inconsistency between classification and localization accuracy. Experiments based on two different datasets demonstrate that each part of our proposed network is efficient, and its overall detection performance is superior to other competing methods.

## Figures and Tables

**Table 1:** Distribution of the Ship dataset.

	Image number	Target number
Training set	800	2834
Test set	202	623

**Table 2:** Effects of each component of CAFC-Net.

Methods	Different variants					
BAM	×	✓	×	✓	✓	✓
FAM	×	×	✓	×	✓	✓
DDM	×	×	×	✓	×	✓
mAP (%)	75.62	78.41	79.86	83.38	85.65	91.18

**Table 3:** Ablation study of the proposed BAM.

Methods	Different variants				
Attention	×	✓	✓	✓	✓
Excitation function	×	×	✓	×	✓
Depression function	×	×	×	✓	✓
mAP (%)	75.62	76.43	77.70	78.21	78.41

**Table 4:** Comparison of AlignConv with other convolution methods.

Methods	mAP (%)	Speed (ms)
Conv	87.48	35
DeformConv	88.68	37
DeformConv v2	89.88	38
AlignConv	91.18	39

**Table 5:** Comparisons with state-of-the-art methods on Ship.

Methods	TP	FP	R (%)	P (%)	F (%)	AP (%)
R^2^CNN	492	82	79.00	85.71	82.22	72.29
RRPN	516	444	82.83	53.75	65.19	75.24
R-DFPN	541	58	86.84	90.32	88.55	81.70
SCRDet	577	38	92.62	93.82	93.22	90.89
R^3^Det	574	110	92.13	83.92	87.83	88.34
CAFC-Net (ours)	572	28	93.26	94.63	93.94	91.18

## Data Availability

The data used to support the findings of this study are included within the article.
